# Bioactive Compounds, Antioxidant, Anti-Inflammatory, Anti-Cancer, and Toxicity Assessment of *Tribulus terrestris*—In Vitro and In Vivo Studies

**DOI:** 10.3390/antiox11061160

**Published:** 2022-06-13

**Authors:** Malik Waseem Abbas, Mazhar Hussain, Saeed Akhtar, Tariq Ismail, Muhammad Qamar, Zahid Shafiq, Tuba Esatbeyoglu

**Affiliations:** 1 Institute of Chemical Sciences, Bahauddin Zakariya University, Multan 60800, Pakistan; wasimchemist229@gmail.com (M.W.A.); zahidshafiq@bzu.edu.pk (Z.S.); 2 Institute of Food Science and Nutrition, Bahauddin Zakariya University, Multan 60800, Pakistan; saeedakhtar@bzu.edu.pk (S.A.); tariqismail@bzu.edu.pk (T.I.); 3 Institute of Food Science and Human Nutrition, Gottfried Wilhelm Leibniz University Hannover, Am Kleinen Felde 30, 30167 Hannover, Germany

**Keywords:** phytochemicals, antioxidant, inflammation, cancer, toxicity, ESI-MS/MS, RP–HPLC, liquitrigenin, myricetin, rutin, bioassay-guided fractionation

## Abstract

*Tribulus terrestris* L. belongs to the family Zygophyllaceae and integral part of various ancient medicinal systems including Chinese, Indian, and European to combat various health ailments. The aim of the present study was to assess the phytochemical constituents, in vitro antioxidant activity using DPPH, FRAP, and H_2_O_2_ assays, in vitro anticancer activity using MTT assay, and in vitro and in vivo anti-inflammatory properties of *T. terrestris*. The acute and sub-acute toxicity of extracts exhibiting most biological potential was examined using murine models. Liquid–liquid partitioning followed by RP–HPLC sub-fraction of crude extract was performed. After that, ESI-MS/MS analysis was done for the timid identification of bioactive metabolites responsible for bioactivities of sub-fractions and HPLC analysis to quantify the compounds using external standards. Among all extracts, *T. terrestris* methanol extract was noted to hold maximum phenolic (341.3 mg GAE/g) and flavonoid (209 mg QE/g) contents, antioxidant activity in DPPH (IC_50_ 71.4 µg/mL), FRAP (35.3 mmol/g), and H_2_O_2_ (65.3% inhibition) assays, anti-inflammatory activities in vitro at 400 µg/mL (heat-induced hemolysis, % inhibition 68.5; egg albumin denaturation, % inhibition 75.6%; serum albumin denaturation, % inhibition 80.2), and in vivo at 200 mg/kg (carrageenan-induced paw edema, % inhibition 69.3%; formaldehyde-induced paw edema, % inhibition 71.3%) and anticancer activity against breast cancer cell (MCF-7) proliferation (IC_50_ 74.1 µg/mL). Acute and sub-acute toxicity studies recorded with no change in body weight, behavior, hematological, serum, and histopathological parameters in treated rats with *T. terrestris* methanol extracts when compared to control group. Fraction B obtained through liquid–liquid partitioning resulted in more bioactive potential as compared to the parent methanol extract. RP–HPLC analysis of fraction B resulted with four sub-fractions (TBTMF1-TBTMF4), wherein TBTMF3 delineated notable bioactive capabilities as compared to other fractions and parent methanol extract. ESI-MS/MS analysis of TBTMF3 resulted with tentative identification of myricetin, rutin, liquitrigenin, physcion, and protodioscin. It can be stated that *T. terrestris* is a potential bearing herb and findings of current study further verify the claims made in ancient medicinal systems. However, after investigation of each identified compound, it must be considered for drug discovery.

## 1. Introduction

More than a billion individuals on the planet suffer from chronic inflammation, which has been linked to a wide range of major health disorders, including trouble in breathing, dermal problems, intestinal issues, and nervous illnesses [1]. Nonsteroidal anti-inflammatory drugs (NSAIDs) and corticosteroids help in easing the discomfort associated with these symptoms [1]. There have been reports that long-term use of anti-inflammatory medicines can affect the correct functioning of many organs in the digestive and excretory systems [2]. Cancer is also a leading cause of mortality in the world, and it is one of the most frequent diseases that may cause death. The International Agency for Research on Cancer has reported 19.3 million new cancer cases and almost 10.0 million cancer related deaths in the year 2020 [3]. Although chemotherapy, radiation, and chemical medicines are extensively used cancer treatment modalities, the costs and risks of treatment outweigh the benefits and constitute a significant worry [4].

As a result, medicines that are both affordable and harmless are necessary for the treatment of inflammatory illnesses. Fruits and vegetables contain several biologically active metabolites that can be used as an alternative to manufactured drugs [5]. Health benefits of plant-based drugs have been well established since the dawn of human civilization. In fact, natural sources have been shown to provide more than 60% of anticancer medicines, including vinblastine and vincristine as well as camptothecin, taxol, podophyllotoxin, and the combretastatins [6]. The discovery of penicillin, morphine, and aspirin, which are semisynthetic acetylated derivatives of natural salicylic acid, demonstrates the significance of plants and fungi in the development of medicine. Morphine possesses anti-inflammatory action, penicillin acts an antibiotic, and aspirin is a cyclooxygenase inhibitor usually given to control fever, pain, inflammation, strokes, and heart attacks owing to its antiplatelet abilities [7]. Conversely, there are limited incidents reported about the toxicity of plant-based products that may appeals the consumers due to their safety, convenience, and affordability [8]. Even then, prior to use of plant-based drugs, substantial scientific evidence-based research is required on the quality, safety, and efficacy parameters [9].

*Tribulus terrestris* L. (*T. terrestris*) belongs to Zygophyllaceae family and widely grown throughout the Mediterranean and subtropics including India, China, Mexico, Spain, Bulgaria, and Pakistan [10]. Goat head, hard thorns, and *Tribulus* are the local names associated with it. *T. terrestris* is considered as vital component of various countries ancient health care systems i.e., India (Ayurveda), Chinese, and Europe [11]. In China, *T. terrestris* fruits are considered helpful in preventing kidney problems, cough, and improves the eyesight [12]. Ayurveda uses the fruits to treat infertility and roots are deliberated as cardiotonic [13]. In Sudan, *T. terrestris* also utilized to prevent or protect inflammation of the glomeruli (nephritis) of the kidney and other inflammatory complaints. It is also used as a diuretic and uricosuric in Pakistan [14]. Traditional medicinal claims about *T. terrestris* are well supported by the pharmacological studies wherein it is widely applied to treat inflammation [13], drying of skin and itchiness [15], heart and blood vessel related ailments [16], carcinomas [17], infections induced by microbes [18], oxidative damage [19], hormonal problems [20], and help to repair muscles [21]. The reported activities were related to saponins, flavonoids, and alkaloids [21,22].

The present work aimed to assess the antioxidant, anti-inflammatory, and anticancer activities of *T. terrestris* extracts using activity-guided approach i.e., solvent–solvent partitioning and reversed phase–high performance liquid chromatography (RP–HPLC). Acute and subacute toxicity tests using animal models will also be performed to evaluate the toxicity of *T. terrestris* different extracts. These studies also examined to further justify the use of *T. terrestris* in ancient medical systems against inflammation and also its non-toxic nature.

## 2. Materials and Methods

### 2.1. Plant Material

*Tribulus terrestris* was collected from the hilly arears of DG Khan, a district of South Punjab, in Pakistan. The Botany department of the Bahauddin Zakariya University (BZU), Multan, Pakistan examined the taxonomy. Collected plant material was cleaned with tap water, allowed to dry under shade, and finally placed inside in an oven (Memmert, Schwabach, Germany) at 40 °C for complete moisture removal. The dried material was crushed in an electric grinder to obtain a fine powder. The obtained powdered material was kept under dark at normal room temperature until extraction.

### 2.2. Solvents and Reagents

Reference standards (ascorbic acid, quercetin, iron (II) sulfate), DPPH, H_2_O_2_, PBS, analytical grade solvents (*n*-hexane, dichloromethane, ethyl acetate, chloroform, methanol, water), and HPLC grade solvents (water, methanol, trifluoracetic acid) were supplied by the local supplier of Sigma-Aldrich (St. Louis, MO, USA).

### 2.3. Animals

Twenty-eight to thirty-five days old experimental animals, i.e., Wistar albino mice (*n* = 40, 25–30 g) and rats (*n* = 40, 200–300 g), were purchased from the Pharmacy Department, BZU, Pakistan and housed in a controlled habitat at 20 °C ± 4 °C with light/dark circle of 12 h. All trials were conducted following preset guidelines of National Research Council (NRC, 1996), Washington, DC, USA. The study was also approved by the internal animal care committee at BZU, Pakistan (approval number ACC-08-2018).

### 2.4. Extraction Procedure

Using a temperature-controlled orbital shaker, the powdered plant material was extracted with *n*-hexane to remove non-polar compounds like fat, followed by successive extractions with dichloromethane, methanol, and 70% aqueous methanol. The extracts were evaporated at 40 °C in a rotary evaporator (Heidolph, Schwabach, Germany) to produce semi-solid extracts and stored at 4 °C in an upright ultralow freezer (Sanyo, MDF-U32V, Osaka, Japan) for further investigations.

### 2.5. Determination of Total Phenolic and Flavonoid Content

All spectrophotometric analysis were performed using a spectrophotometer (V-3000; VWR, Darmstadt, Germany). Total phenolic content was determined spectrophotometric using Folin-Ciocalteu assay according to Singleton and Rossi (1965) [23]. Results were expressed as mg gallic acid equivalent/gram on dry weight (mg GAE/g dw) basis. Total flavonoid content was determined following the aluminum chloride assay according to Pękal and Pyrzynska (2015) [24]. Results were expressed as mg quercetin equivalent/gram on dry weight (mg QE/g dw) basis.

### 2.6. Determination of Antioxidant Activity Using Spectrophotometric Assays

The 2,2-diphenyl-1-picrylhydrazyl (DPPH) scavenging capability of *T. terrestris* crude extracts (1 g/20 mL) was tested according to Alara et al. (2019) at 517 nm [25]. As standards, quercetin and ascorbic acid were used, with methanol as a blank. The percentage inhibition was deliberated using the formula below, and the results were expressed as IC_50_:

% inhibition = 100 × (Ac − As)/Ac

where Ac = absorption of the control and As = absorption of the plant sample.

Similarly, the ferric reducing antioxidant capacity (FRAP) of *T. terrestris* crude extracts (1 g/20 mL) was evaluated at 593 nm according to Zahin et al. (2010) [26] using iron sulfate for standard calibration. The results were compared to ascorbic acid and quercetin and represented as mmol quercetin or ascorbic acid mmol/g on a dry weight basis.

The capacity of *T. terrestris* crude extracts (1 g/20 mL) to scavenge hydrogen peroxide (H_2_O_2_) was determined at 593 nm following the method of Ruch et al. (1989) [27]. Positive controls included ascorbic acid and quercetin, with phosphate buffer serving as blank sample. The following formula was used to compute the percentage of H_2_O_2_ inhibition:

H_2_O_2_ scavenging activity (%) = 100 × (Ab − As)/Ab

where Ab = absorption of the blank and As = absorption of the test sample.

### 2.7. In Vitro Anti-Inflammatory Activity

#### 2.7.1. Membrane Stabilization Assay

The activity was performed to investigate the ability of *T. terrestris* crude extracts against heat-induced hemolysis. The activity was performed on blood samples taken from the cubital vein of healthy individuals (resident in Karachi, Sindh, Pakistan). The guidelines of the International Federation of Blood Donor Organizations (IFBDO) were followed, while collecting the blood samples and also authorized by the Bioethical Committee of Bahauddin Zakariya University, Multan, Pakistan (approval number Reg. No. 10–20). This work was also carried out in conformity with the Helsinki declaration. Blood samples were centrifuged using a laboratory centrifuge (Model 800b, Jintan, China) for 5–7 min at 2000 rpm, washed using normal saline, and reconstituted as 10% suspension with isotonic buffer solution (10 mM sodium phosphate buffer, pH 7.4; *v*/*v*) [28,29]. One milliliter of *T. terrestris* sequential crude extracts, and concentrations of 100 µg/mL, 200 µg/mL, 300 µg/mL, and 400 µg/mL, were added to red blood cell suspension (1 mL, 10%). The incubation of the reaction mixture was carried out at 50 °C for 30 min, allowed to cool under dark at room temperature, and centrifuged for 5–7 min at 2500 rpm to take absorbance using a spectrophotometer at 560 nm. Phosphate buffer was taken as control and diclofenac sodium as standard drug. Results were presented as % inhibition and the following equation was used for calculation.

% Inhibition of denaturation = 100 × (Ac − As)/Ac

where AC = absorption of the control, and AS = absorption of the plant sample.

#### 2.7.2. Egg Albumin Denaturation Assay

The egg albumin denaturation inhibition potential of *T. terrestris* sequential crude extracts were determined according to Mizushima and Kobayashi (1968) [30] with slight modifications. *T. terrestris* sequential crude extracts (2 mL) of varying strengths (100, 200, 300, and 400 µg/mL) were added to 0.2 mL of egg albumin and 2.8 mL of phosphate buffer saline (pH = 6.5). After that, incubation (37 °C, 25 min), heating (70 °C for 35 min), and cooling of the reaction mixture was carried out under dark at room temperature. The absorbance was measured at 660 nm. Phosphate buffer was taken as control and diclofenac sodium as standard drug. The results were recorded as percentage inhibition using the above-mentioned equation in Section 2.8.2.

#### 2.7.3. Bovine Serum Albumin Denaturation Assay

The activity was performed using Sakat et al. (2010) [29] method with minor changes. In this experiment, the reaction mixture (0.5 mL) was made by mixing 50 µL *T. terrestris* sequential crude extracts (100–400 µg/mL) with bovine serum albumin (450 µL) followed by incubation (25 °C, 30 min). Second incubation was performed after addition of phosphate buffer (0.5 mL, pH = 6.5) to the reaction mixture for twenty minutes at 65 °C. The absorbance of the reaction mixture was taken at 660 nm but before that it was allowed to stand for more than thirty minutes under dark. The results were recorded as percentage inhibition using the above-mentioned equation in Section 2.8.2.

### 2.8. In Vivo Anti-Inflammatory Activity

#### 2.8.1. Inhibition of Carrageenan-Induced Paw Edema in Wistar Rats

Carrageenan-induced paw inflammation assay was employed to assess the pain reliving capabilities of *T. terrestris* sequential crude extracts according to Morris (2003) [31] after few changes. In the current study, we made eight groups and each group comprised of 5 animals (*n* = 5). Animals in group one were provided with normal saline and named as control group. Animals in group two were given standard indomethacin as 100 mg/kg, body weight (b.w.), and named as positive control. Rats in group three and four were fed with the dichloromethane extract of *T. terrestris* as 100 mg/kg and 200 mg/kg, separately. Rats in group 5 and 6 were fed with 100% methanolic extract of *T. terrestris* as 100 mg/kg and 200 mg/kg, respectively. Likewise, rats in group seven and eight were fed with 70% methanolic extract of *T. terrestris* as 100 mg/kg and 200 mg/kg, separately. Importantly, initial size of paw circumference of every rat was noted. Right after half hour of extracts administration, animals were injected with carrageenan into the plantar aponeurosis surface of the right hind paw. After that, any change in paw linear circumference was noted after 0 h, 1 h, 2 h, and 3 h employing Plethysmometer (UGO-BASILE 7140, Comerio, Italy). The rise in paw circumference was deliberated as a sign of swelling.

#### 2.8.2. Inhibition of Formaldehyde-Induced Hind Paw Edema in Albino Mice

Formaldehyde-induced hind paw edema assay was used to examine the anti-inflammatory potential of *T. terrestris* different extracts in mice adopting the method of Brownlee after minor changes [32]. The Albino mice were put into eight groups of five (*n* = 5). Each group received the same treatment as mentioned in the Section 2.8.1. After a half hour of extracts administration, formaldehyde (100 µL, 4%) was injected into the plantar aponeurosis of each mouse’s right paw, and change in paw circumference was recorded after 0, 3, 6, 12, and 24 h.

### 2.9. Acute and Sub-Acute Toxicity Assessment

In vivo toxicity assessment of *T. terrestris* extracts was conducted adopting the guidelines of Organization for Economic Cooperation and Development (OECD) 407 and 423 for acute oral toxicity tests [33,34]. Rats were provided with clean water and food (ad libitum) for 24 h before and after the trial. All animals were fed with *T. terrestris* 100% methanolic at doses 2000 and 3000 mg/kg (per oral) for 14 days. Just after 1 h of extract administration, an examination of each animal was performed periodically after 4 h, 8 h, 12 h, and 24 h for any change in behavior. Change in body weight of each animal was evidenced after 7 days and then blood samples were taken to analyze hematological and biochemical parameters. After that, rats were sacrificed and their organs, including liver, heart, and kidney, were isolated to perform histopathological examination.

For chronic toxicological studies, all rats were starved for at least 2 h and then orally fed with 500 and 1000 mg/kg of 100% methanolic *T. terrestris* extract for 28 days. Just after 1 h of extract administration, an examination of each animal was performed periodically after 4, 8, 12, and 24 h for any change in behavior. Change in body weight of each animal was evidenced after 7 days and then blood samples were taken to analyze hematological and biochemical parameters. After that, rats were sacrificed and their organs, including liver, heart, and kidney, were isolated to perform histopathological examination.

### 2.10. Histopathological Analysis

Fresh portions of kidney, liver, and heart were taken from the rats of treated and normal group. The collected parts of the organs were cut and then fixed in the solution of formalin (10%). After that, dehydration of the fixed samples was performed with dilution series (60–100%) of alcohol and inserted in paraffin. The blocks of organs fixed in paraffin were cut into pieces with 4 µm thickness. These sections were inspected using light microscope to see histopathological changes (Nikon Eclipse 50i, Tokyo, Japan) after staining with hematoxylin and eosin and at the end photomicrographs were taken.

### 2.11. Liquid–Liquid Partitioning of the Active Crude Extract

The methanolic extract of *T. terrestris*, which exhibited better biological activities and also proved nontoxic in animal studies, was subjected to solvent–solvent partitioning to obtain more active fractions. In detail, in a beaker with 15 mL of water, 100% methanolic extract was shaken vigorously and then 15 mL chloroform was added after 30 min and the mixture was poured into a separator funnel. The next day, two layers were visible: Aqueous at the top and chloroform at the bottom. Next, chloroform layer was recovered and named as fraction A. After that, aqueous fraction was again added with 15 mL of ethyl acetate and allowed stand for whole night. The next day, again two layers were visible: ethyl acetate at the top and aqueous at the bottom. Both layers were recovered and named as fraction B (ethyl acetate) and fraction C (water). Importantly, all the recovered fractions were evaporated under reduced pressure at 40 °C on rotary evaporator (Heidolph, Schwabach, Germany).

### 2.12. Reversed-Phase (RP)-HPLC of Fraction B and Its Sub-Fractions

#### 2.12.1. Analytical Measurements of Fraction B (Ethyl Acetate)

Solvent–solvent partitioned fraction B (ethyl acetate) of *T. terrestris* methanolic extract showed considerable biological potential in contrast to fraction A (chloroform) and C (water). Considering the bioassay-guided approach, fraction B was further partitioned in order to have more active fractions using RP–HPLC. Fractionation was performed on an HPLC system with a diode array detector (DAD) (1100/1200 series, Agilent, Waldbronn, Germany) with an analytical column (Zorbax-SB-C18, 4.6 × 150 mm, 5 μm, Agilent, Waldbronn, Germany) [35]. The temperature of the column was maintained at 30 °C. Fraction B (ethyl acetate) was dissolved in methanol (5 mg/mL) and filtered using 0.45 mm syringe filter. Mobile phase was composed of 0.1% triflouroacetic acid (A), and acetonitrile with 0.1% triflouroacetic acid (B). Ten microliters of the filtered sample were loaded onto the HPLC column and the flow rate was set at 0.5 mL/min, whereas the gradient elution system was as follows: 15% B in 0–5 min, 15–30% B in 5–10 min, 30–70% B in 10–25 min, 70–85% B in 25–27 min, and 100 % B in 27–30 min. Chromatograms were recorded at 280 nm.

#### 2.12.2. Semi-Preparative Chromatography of Fraction B (Ethyl Acetate)

Seventy microliters sample (1 g/10 mL) was loaded onto the semi-preparative column (Zorbax-SB-C18, 25 × 250 mm, 5 μm particle size, Agilent, Waldbronn, Germany) keeping all the other parameters same as mentioned in the previous section. Total four sub-fractions were retrieved named TBTMF1, TBTMF2, TBTMF3, and TBTMF4 from fraction B (ethyl acetate) of *Tribulus terrestris* 100% methanolic extract.

### 2.13. LC-ESI-MS/MS Analysis of Sub-Fraction TBTMF3

All fractions obtained using semi-preparative RP–HPLC were evaluated for their in vitro bioactive potential wherein only TBTMF3 outlined noteworthy activities which was further analyzed on LC-ESI-MS/MS (LTQ XL, Thermo Electron Corporation, Walthan, MA, USA) for the tentative identification of bioactive metabolites according to Steinmann and Ganzera. (2011) [35]. The structures of the compounds were identified using online software and compared with published literature (www.chemspider.com, accessed on 4 October 2021).

### 2.14. Quantification of Compounds in Sub-Fraction TBTMF3 Using Analytical HPLC-DAD

A hundred milligrams of solidified sub-fraction TBTMF3 was dissolved in 1 mL methanol and standards, including myricetin, rutin, and protodioscin (each 250 μg/mL), were also prepared in methanol. Following that, the samples were centrifuged for 10 min at 14,000 rpm to collect the supernatant. After filtration using a syringe filter, 100 μL sample and standards were injected into the HPLC system for analysis. All other parameters were the same, as mentioned in Section 2.12.1. The identification was performed by comparing the UV spectra and retention times with those of authentic standards.

### 2.15. Statistical Analysis

This study’s data are provided as mean (SEM) of three measuremnets. ANOVA was used to compare the differences between the control and treatment groups, and Dunnett’s test was run using Graph pad prism (Graph Pad Software, San Diego, CA, USA, http://www.graphpad.com, accessed on 3 March 2021).

## 3. Results

### 3.1. Extraction Efficiency, Phytochemical Contents, and In Vitro Antioxidant Activity of T. terrestris Extracts

*Tribulus terrestris* L. powder was initially defatted using *n*-hexane. After that, the residue on the filter paper was extracted with dichloromethane for 48 h under stirring and again residues fractioned using methanol and 70% aqueous methanol. The methanol extraction offered maximum yield (1.23%), followed by 70% aqueous methanol (0.62%) and dichloromethane (0.12%). Similarly, total phenolic contents were recorded higher in methanol extract of *T. terrestris* followed by 70% aqueous methanol, and dichloromethane extracts as 341.3 mg gallic acid equivalent (GAE)/g, 92.9 mg GAE/g, and 4.40 mg GAE/g, respectively. Likewise, total flavonoid contents were found maximum in methanol extract (209 mg quercetin equivalent QE/g) and lowest in dichloromethane extract (2.20 mg QE/g).

*T. terrestris* methanol extract showed considerable antioxidant activity in DPPH (IC_50_ of 71.4 µg/mL), FRAP (35.3 Fe mmol/g), and H_2_O_2_ (% inhibition 65.3) assays in contrast to 70% aqueous methanol and dichloromethane extracts outlined intermediate to non-substantial activity (Table 1).

### 3.2. In Vitro Anti-Inflammatory Activity of T. terrestris Sequential Crude Extracts

Red blood cell membrane stabilization, serum, and egg albumin denaturation tests were used to examine the in vitro anti-inflammatory activities of *T. terrestris* methanol, 70% aqueous methanol, and dichloromethane extracts. Table 2 outlines the results of crude extracts at varying concentrations i.e., 100, 200, 300, and 400 µg/mL. *T. terrestris* methanol extract displayed notable membrane stabilization capabilities by inhibiting heat-induced hemolysis (% inhibition 68.5 at 400 µg/mL, *p* < 0.001) followed by intermediate activity of 70% aqueous methanol extract (% inhibition 35.5 at 400 µg/mL, *p* < 0.01) when compared to phosphate buffer, i.e., control. The standard drug diclofenac sodium displayed potent anti-inflammatory activity by inhibiting the heat-induced hemolysis (% inhibition 89.3 at 400 µg/mL, *p* < 0.0001).

In serum, albumin denaturation assay methanol extract of *T. terrestris* induced a noticeable inhibition of 80.2 % at 400 µg/mL (*p* < 0.001) in a concentration-dependent manner after diclofenac sodium offered potent inhibition of 97.8% (*p* < 0.0001) at same dosage when compared phosphate buffer (control). At the same concentration, 70% aqueous methanol and dichloromethane extracts evidenced moderate (48.3 % at 400 µg/mL, *p* < 0.01) to no activity, respectively. In our egg albumin denaturation experiment, methanol extract of *T. terrestris* again induced significant inhibition of 75.6% at 400 µg/mL (*p* < 0.001) followed by moderate anti-inflammatory effects of 70% aqueous methanol observed with 43.2% inhibition at same concentration in contrasted to phosphate buffer i.e., control. At identical concentration, standard drug diclofenac sodium presented significant inhibition of 91.5% (*p* < 0.0001).

### 3.3. In Vivo Anti-Inflammatory Activity of T. terrestris Sequential Crude Extracts

**Figure 1:**
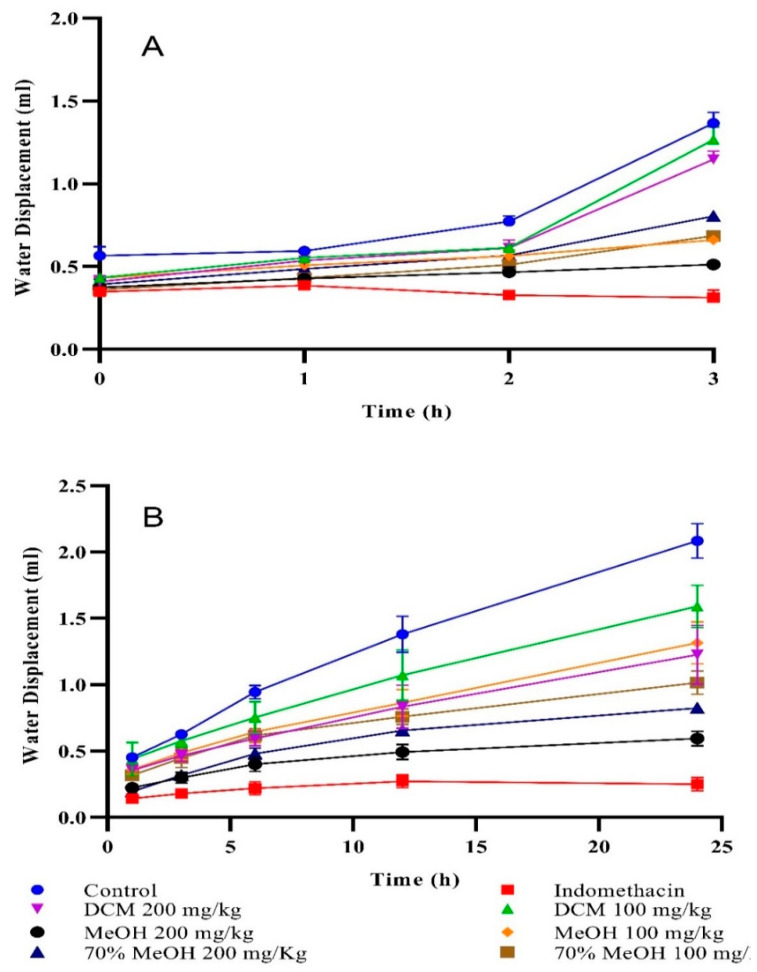
(**A**) Carrageenan-induced and (**B**) formaldehyde-induced paw edema inhibition. Values are mean ± SEM of three readings. The standard drug (Indomethacin) exhibited potent inhibition of 86.3% (*p* < 0.0001) and 89.3% (*p* < 0.0001) against carrageenan and formaldehyde intoxicated paw edema at 100 mg/kg, respectively when compared to control (normal saline = 0% inhibition). On the other hand, dichloromethane and 70% aqueous methanol extracts induced moderate to non-substantial inhibition in both assays.

*T. terrestris* dichloromethane, methanol, and 70% aqueous methanol extracts were examined for in vivo anti-inflammatory activities against carrageenan (Figure 1A) and formaldehyde-induced paw edema (Figure 1B) in rats and mice, respectively. Methanol extract outlined significant inhibition of carrageenan- (69.3%, *p* < 0.001) and formaldehyde- (71.3%, *p* < 0.001) induced paw swelling at 200 mg/kg b.w., after 3 and 24 h, separate when compared to control.

### 3.4. In Vitro Anticancer Activity of T. terrestris Crude Extracts

A series of *T. terrestris* crude extracts (dichloromethane, methanol, and 70% aqueous methanol) were examined for their antineoplastic activity against different cancer cell lines including MCF-7 (breast cancer cell line), HeLa (cervical cancer cell line), SK-OV-3 (ovarian carcinoma), and NCI-H522 (lung cancer) using MTT assay (Table 3). Only methanol extract strongly inhibited breast and ovarian cancer cell multiplication with an IC_50_ of 74.1 µg/mL and 89.4 µg/mL, whereas 70% aqueous methanol and dichloromethane had no impact.

### 3.5. In Vivo Acute and Subacute Toxicity Assessment

*T. terrestris* methanol extract exhibited excellent biological potential in radical scavenging, anti-neoplastic, and anti-inflammatory tests, whereas other two extracts (70% aqueous methanol and dichloromethane) exerted moderate to no activity in aforementioned assays (Table 1, Table 2 and Table 3; Figure 1a,b). Therefore, only methanol extract was selected for further analysis of in vivo acute and subacute toxicity assessment. In the present study, body weight and behavior of experimental rats was found in order and no death was recorded throughout a 14-day acute toxicity experiment. Added to that, organs weight (liver, heart, kidney) of experimental rats were also normal (Table 4). Therefore, the LD_50_ of *T. terrestris* methanol extract was supposed to be around or more than 3000 mg/kg.

Moreover, sub-acute toxicity trial (28-days) of *T. terrestris* methanol extract was also conducted, and the results are shown in Table 4, which demonstrate that the weight of the rats treated with 500 mg/kg and 1000 mg/kg of *T. terrestris* methanol extract did not differ significantly from that of the control group (receiving normal saline). Treatment-group rats had the same heart, kidney, and liver weight as those in the control group.

Table 4 shows the hematological and serum parameters for the acute and subacute investigations. The findings suggest that animals fed with *T. terrestris* methanol extract for 14 days had a modest rise in hematological and serum parameters, although these values were still within normal ranges. *T. terrestris* methanol extract, on the other hand, exhibited identical effects in a 28-day trial of sub-acute toxicity, and did not cause any changes in hematological parameters. Apparently, there was no change in the hematological and serum profile between normal and treated rats in acute and subacute tests, which shows that the *T. terrestris* methanol extract had no detrimental impact on the liver or kidney.

**Figure 2:**
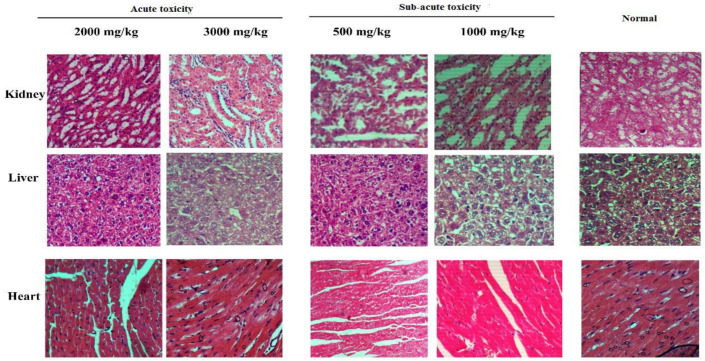
Histopathology results of acute and subacute toxicity of *T. terrestris* methanol extract.

In Figure 2, histopathological sections from the heart, liver, and kidneys of acute and subacute toxicity evaluation are shown. A closer look under the microscope revealed no abnormalities in terms of histopathology among rats treated with *T. terrestris* methanol extract when compared to the control group.

### 3.6. Bioassay-Guided Approach

#### 3.6.1. Liquid–Liquid Partitioning of Crude Extracts

The liquid–liquid partitioning of methanol extract yielded three fractions, i.e., chloroform (fraction A), ethyl acetate (fraction B), and water (fraction C) as described in Section 2.11. According to the preceding sections, fraction B (ethyl acetate) has higher antioxidant properties than the parent methanol extract. However, fractions C and A showed little to no activity (Table 5). Moreover, fraction B also displayed notable anti-inflammatory activities in membrane stabilization assay (74.1%, *p* < 0.001), egg albumin denaturation inhibition (77.9%, *p* < 0.001), and serum albumin denaturation inhibition (83.5%, *p* < 0.001) at 400 μg/mL when compared to the control i.e., phosphate buffer. However, fractions A and C presented moderate to no activity, respectively. Similarly, in the anticancer experiments, fraction B was shown to have the greatest inhibitory impact on breast and ovarian cancer cell growth, with IC_50_ values of 65.2 g/mL and 81.3 g/mL, respectively, but no activity was found against cervical and lung cancer cell lines (Table 5). Furthermore, fractions A and C were shown to have no significant effect against any of the cancer cell lines tested.

#### 3.6.2. Preparative HPLC Sub-Fractionation of the Liquid–Liquid Partitioned Fraction B

Fraction B (ethyl acetate) of *T. terrestris* methanol extract presented excellent bioactive properties in the aforementioned section, and was further separated using RP–HPLC and obtained four sub-fractions TBTMF1–TBTMF4, as described in Section 2.12. All retrieved sub-fractions were examined for their radical scavenging, inflammation reduction, and anti-tumor potential (Table 6). Consequently, TBTMF3 demarcated more antioxidant potential in DPPH (IC_50_ 41.9 µg/mL), FRAP (49.1 mmol/g), and H_2_O_2_ (71.2% inhibition) assays in contrasted to their liquid–liquid partitioned fraction B and parent methanol extract. The activity was found in accordance with standard ascorbic acid and quercetin (Table 1). Correspondingly, TBTMF3 also displayed notable anti-inflammatory activities in membrane stabilization assay (76.1%, *p* < 0.001), egg albumin denaturation inhibition (81.9%, *p* < 0.001), and serum albumin denaturation inhibition (85.2%, *p* < 0.001) at 400 μg/mL when compared to control i.e., citrate buffer in concentration reliant mode (Table 2). In addition to this, the IC_50_ of TBTMF3 against breast cancer cells was recorded as 43.2 µg/mL, compared to 65.2 µg/mL for the liquid–liquid partitioned fraction (fraction B) and 74.1 g/mL for the parent extract (methanolic extract) (Table 3).

### 3.7. ESI-MS/MS and HPLC Analysis of TBTMF3 Fraction

**Figure 3:**
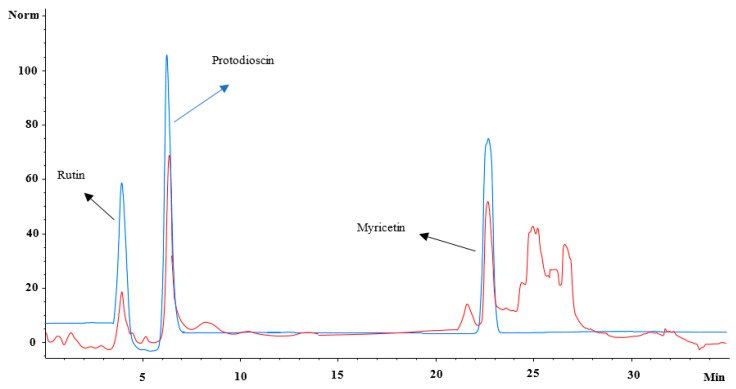
Overlay of HPLC-chromatograms of standards (blue) and the TBTMF3 fraction (red) recorded at 280 nm by HPLC-DAD.

TBTMF3 outlined significant radical scavenging potential i.e., in accordance with quercetin and ascorbic acid, notable inflammation reduction abilities in contrasted to control group (phosphate buffer), and inhibited the multiplication of breast cancer cell in previous sections. Therefore, TBTMF3 was analyzed by ESI-MS-MS in positive mode in order to have tentative identification of bioactive metabolites. The analysis unveiled the presence of myricetin (MS, 317; MS/MS [M+H], 317, 299, 272), liquitrigenin (MS, 256; MS/MS [M+H], 255, 237), physcion (MS, 283; MS/MS [M+H], 268, 267, 239, 211), protodioscin (MS, 1048; MS/MS [M+H], 1048, 1031, 998, 741), and rutin (MS, 610; MS/MS [M+H], 465, 333), confirmed by comparing with the previous published literature by Gates and Lopes, (2012) [36], Rahman et al. (2018) [37], Chen et al. (2016) [38], Ivanova et al. (2016) [39], and Said et al. (2016) [40], respectively. The main compound in the RP–HPLC sub-fraction TBTMF3, rutin, protodioscin, and myricetin, was quantified using external standards at 280 nm. All these compounds were quantified as 2.19 µg/mg, 11.2 µg/mg, and 4.20 µg/mg, respectively (Figure 3, Table 7).

## 4. Discussion

In the present study, *T. terrestris* methanol extract offered maximum affinity towards total phenolic and flavonoid contents. Hitherto, ethanol extract of *T. terrestris* was noted with total phenolic and flavonoid contents as 41.2 mg catechol equivalent (CE)/g and 601 mg QE/g, notably higher than acetone extract [41], in alliance with the findings of current investigations. The outcomes of the present study are also supported by the results of Naz et al. (2017) [42] wherein methanol extract was calculated with prominent amount of total phenolic (426 mg GAE/g) and flavonoid (371 mg QE/g) contents. Moreover, *T. terrestris* methanol extract exhibited excellent antioxidant activity as compared to 70% methanol and dichloromethane extracts. This might be attributed in part to the existence of polar functional groups in the polyphenolic structures, believed to have radical scavenging potential. Earlier research had demonstrated that phytocompounds were involved in stabilizing reactive oxygen species by donating hydrogen ions and terminated free radicals initiated deleterious chain events, both of which are detrimental to health [43].

Earlier, in the DPPH assay, the IC_50_ of *T. terrestris* methanol extract was noted as 92 μg/mL whereas standard ascorbic acid and rutin displayed IC_50_ of 5 and 18 μg/mL individually [42]. In the present study, it can be stated that higher antioxidant activity of *T. terrestris* methanol extract in all three assays is due to the presence of the phenolic and flavonoid contents. Lately, a direct relation between total phenolic contents and antioxidant activity has been shown by Arshad et al. (2020) [44]. In brief, the leaves of methanol extract of *T. terrestris* outlined 73% inhibition in the DPPH assay as compared to 52% inhibition of root extract, which also offered more affinity towards total phenolic (723 mg GAE/g) and flavonoid (476 mg QE/g) extraction than root extract recorded with total phenolic and flavanoid contents of 235 mg GAE/g, and 93 mg QE/g, respectively.

Anti-inflammatory medicines have the ability to impart notable stabilization effects towards human red blood cell membrane against hypotonicity-induced lysis [45]. In the present study, *T. terrestris* methanol extract displayed notable potential in stabilizing human red blood cell membrane by inhibiting the heat-induced hemolysis, i.e., consistent with its phytochemical and antioxidant potential. The findings are in alliance with the previous report of Naz et al. (2017) [42] wherein *T. terrestris* methanol extract demonstrated noteworthy membrane stabilization activity by inhibiting heat-induced hemolysis (% inhibition 69.0 at 200 µg/mL) in a dose-dependent mode. In another study, ethanol extract of *T. terrestris* also reported to induce moderate membrane stabilization effects in a dose-dependent mode [46]. The concentration reliant antispasmodic activities of standard anti-inflammatory drugs, i.e., salicylic acid and phenylbutazone, has also been observed previously [30].

Protein denaturation is a primary source of inflammation, and reliable studies described previously support this connection [47,48]. In our study, *T. terrestris* methanol extract efficiently inhibited the serum albumin denaturation and egg albumin denaturation. The findings of Naz et al. (2017) [42] found *T. terrestris* methanol extract effective against denaturation of serum albumin (72% inhibition at 200 µg/mL), which braced the results of our current investigation. It can be suggested that anti-inflammatory potential portrayed by *T. terrestris* methanol extract might be attributed by the total phenolic and flavonoid contents quantified earlier in this study. The presence of phytochemicals like anthocyanins, stilbenes, phenolic acids, tannins, flavonoids, alkaloids, and steroids in plants were reported to be the key factor behind their anti-inflammatory potential [49]. *Calluna vulgaris* ethanol extract was reported to hold more radical scavenging activity (64.2% inhibition of thiobarbituric acid reactive substances (TBARS) content; 80.0% inhibition of DPPH) than *Ferula hermonis* and *T. terrestris* ethanol extracts. The membrane stabilization potential was also found in alliance with antioxidant activities, wherein *Calluna vulgaris* ethanol extract showed higher anti-inflammatory activity followed by *Ferula hermonis* and *T. terrestris* ethanol extracts [46]. It is believed that inflammation reduction potential of natural products and standard drugs might be linked to the erythrocyte membranes following change or modification in cell surface charges. Phytochemical constituents having radical scavenging potential suggested to be involved in stabilizing red blood cell membranes by inhibiting the physical collision with agents capable for aggregation or by encouraging dispersal of similar charges by means of mutual repulsion [50]. Secondary plant metabolites like flavonoids and saponins were reported to involve in the stabilization of lysosomal membrane during in vitro and in vivo studies. On the other hand, saponins and tannins were also reported to stabilize erythrocyte membranes and biological macromolecules ascribed by their excellent ability to bind cations [28].

For the anti-inflammatory potential of *T. terrestris*, when further evaluated using an in vivo study, methanol extract outlined maximum inhibition against carrageenan and formaldehyde intoxicated edema. Previously, *T. terrestris* methanol extract was reported to avert inflammation in rats (70% percent inhibition) induced by carrageenan which was notably higher than chloroform extract (61.9 percent inhibition) and lower than standard drug indomethacin (82.3 percent inhibition) [13]. Anti-inflammatory potential of this traditional herb was also reported by Sudheendran et al. (2017) [51] wherein root and fruit decoction of *T. terrestris* outlined noteworthy inhibition against carrageenan intoxicated swelling in rats, but activity was a little lower than standard drug diclofenac sodium. Methanol extract of *T. terrestris* in another study was reported to show inflammation reduction capabilities in rats comparable to the standard drug indomethacin in carrageenan intoxicated edema model. It was also reported that activity was due to the presence of secondary metabolites (saponins, tannins, flavonoids), also indented in the same study [52]. The results of the present study are also in agreement with the findings of Baburao et al. (2009) [53], which recorded methanol extract of *T. terrestris* to avert carrageenan-induced paw inflammation in rats comparable with standard drug, i.e., diclofenac sodium.

Plant polyphenols can be considered as safer anti-carcinogenic macromolecules because of their cytoprotective activities in normal cells and their simultaneous cytotoxic response toward malignant cells [54]. *T. terrestris* methanol extract in the previous section linked with inhibition of carcinogens such as superoxide, hydrogen peroxide, and hydroxyl radicals possessed anti-cancer activity against breast cancer cell line. Breast cancer cell line suppression by methanol extract is consistent with Bedir et al.’s (2002) [55] report, wherein *T. terrestris* steroidal saponins component were also found as active against the proliferation of breast (IC_50_ of 6.0 µg/mL) and ovarian (IC_50_ of 8.2 µg/mL) cancer cell lines. In another experiment, *T. terrestris* crude methanol extract and its saponins-rich fraction reported to induce strong inhibition against the proliferation of breast cancer cells (MCF-7) but found less toxic against normal breast cell lines (MCF-10A) [39].

Before moving on to clinical trials, safety evaluation experiments give critical data for the toxicity of herbal treatments. Despite the fact that herbal extracts have been shown to have a variety of bioactivities and have the potential for a wide range of applications, the possible adverse effects of herbal extracts are frequently overlooked. Furthermore, it is a valuable measure for determining the therapeutic index of medications and xenobiotics [56,57]. *T. terrestris* methanol extract was subjected to acute and subacute toxicity assessment because of its higher biological potential in comparison to other tested extracts i.e., 70% methanol and dichloromethane. As we mentioned earlier, the extract was safe to the animals during both toxicity studies. In the recent past, *T. terrestris* methanol extract, when dispensed at the rate between 2.00–10.0 g/kg, b.w. for two weeks, was reported to induce no changes in body weight, eating or drinking behavior, or mortality among Swiss mice [58]. The acute and subacute toxicity of *Amrutadi churna*, an Ayurvedic poly herbal formulation made of three herbs (*Tribulus terrestris*, *Emblica officinalis*, *Tinospora cordifolia*), was evaluated in rats. Toxicologically, when rats were even fed with higher dose of *Churna* (5000 mg/kg) it caused neither death nor severe toxicity in rats [59], which outlines the non-toxic nature of traditional herbs. Cisplatin toxicity was prevented in mice when *T. terrestris* fruit extract was administered orally at levels of up to 500 mg/kg body weight [60]. In rat model, *T. terrestris* fruit methanol extract, when examined for toxicity on gastric mucosa, was reported to induce no ulcers at 200 mg/kg, but on the other hand indomethacin, a well-known anti-inflammatory drug, induced ulcers [52]. The dilation of Bowman’s capsule, medullar congestion, and dilatation of collecting tubules alongside reduced body and kidney weight were all seen in the cisplatin-treated mice group. For the first four days of treatment with *T. terrestris* fruit extract these values were seen within the normal range. Lately, *T. terrestris* hydroethanolic fruit extract, when dispensed at the rate of 100 mg/kg b.w., for 30 days, altered the negative effects on hematological parameters in male Wistar rats induced by acephate. Recently, hematology, serum biochemistry, and organ histology studies demonstrated that *Amrutadi churna* containing *Emblica officinalis*, *T. terrestris*, and *Tinospora cordifolia* did not cause hazardous effects at tested dose levels of 500 and 1000 mg/kg b.w, but it caused a mild hyperbilirubinemia with no liver damage [59].

Activity guided fractionation is an effective and successful technique in identification, isolation, and purification of anti-tumor, anti-fertility, and anti-bacterial plant metabolites [61,62,63]. In our study, liquid–liquid portioned fraction B showed more biological potential than crude methanol extract. Correspondingly, RP–HPLC sub-fraction 3 (TBTMF3) of fraction B showed in vitro antioxidant, anti-inflammatory, and anticancer potential more than other sub-fractions and fraction B. Recently, bioassay-guided fractionation resulted in increased biological potential when compared to crude extracts [64,65,66].

It can be stated that bioactive potential of TBTMF3 is due to the presence of aforementioned compounds, as in preceding studies, protodioscin, rutin, myricetin, and liquitrigenin were reported to exhibit anti-inflammatory activities [67,68,69,70]. Myricetin was also found to be effective against human breast cancer (MCF-7) cells’ proliferation by suppressing p21-activated kinase 1 via downstream signaling of the β-catenin pathway [71]. In another study, myricetin was cited to induce anti-tumor activity against MCF-7 breast cancer cells wherein it inhibited the apoptotic BRCA1-GADD45 pathway [72]. Liquiritigenin, on the other hand, has been shown to prevent carcinogenesis in triple-negative breast cancer cells (MDA-MB-231 and BT549) by enhancing breast cancer 1 (BRCA1) transcriptional activity and reducing DNA methyltransferase (DNMT) activity [73]. Furthermore, it has been observed that rutin can help to reestablish chemo-sensitivity in human breast cancer cell lines [74]. In another study, rutin lead for the treatment of triple negative breast cancer (MDA-MB-231/GFP) orthotopic xenografts in a mouse model [75]. As a result, it is reasonable to conclude that the anticancer activity of TBTMF3 against the breast cancer cell line is due to the synergistic action of all of the bioactive metabolites listed above. Previously, Dincheve et al. (2008) [76] quantified protodioscin and rutin as 567.9 µg/g dw and 762.9 µg/g dw in fruits, 10,004 µg/g dw and 2037 µg/g dw in leaves, 193.3 µg/g dw, 92.4 µg/g dry weight (dw) in roots, respectively. In another study, protodioscin and rutin were also quantified from *T. terrestris* plant extracts of varying origins. In brief, protodioscin contents were calculated as 3.04, 6.43, 17.84, 14.68, 20.48 mg/g dw from *T. terrestris* grown in Hungary, Turkey, Vedrare wild growing, Vedrare in culture, and Plovdiv wild growing, respectively. Likewise, rutin contents were recorded as 1.02, 1.51, 0.34, 0.11, 0.11 mg/g dw from *T. terrestris* grown in Hungary, Turkey, Vedrare wild growing, Vedrare in culture, and Plovdiv wild growing, respectively [77]. The amount detected in previous studies is higher than recorded in our experiment and the difference might be due to different geography, growing conditions, and methods employed in sample preparation and identification. Previously, rutin contents calculated from *T. terrestris* of six different localities or regions having different phenotype and chemotype were different from each other. *T. terrestris* grown in shady areas were reported to identified with thirteen flavonoids as compared to *T. terrestris* grown in sunny areas identified with four flavonoids [78].

## 5. Conclusions

The outcomes of the present study demonstrate the radical scavenging capabilities, anti-inflammatory abilities in both laboratory trials and murine models, and anti-tumor potential of *T. terrestris* extracts, supporting the use of this herb in ancient health care systems in various parts of the world. Essentially, acute (14-days) and sub-acute (28-days) toxicity trials also confirmed the practical safety of *T. terrestris* because body weight, organs weight, behavior, hematological, and serum parameters of treated rats were found in accordance with rats of control group. Added to that, no death was observed during both toxicity trails. As result, it can be safely suggested that lethal dose i.e., LD_50_ of *T. terrestris* methanol extract, is greater than 3000 mg/kg. Bioassay-guided fractionation resulted with increased activity like fraction B exhibited more bioactive potential than parent methanol extract and similarly RP–HPLC sub-fraction TBTMF3 outlined more activity than fraction B and parent methanol extract. It is a well-established fact that in a raw extract, activity of bioactive metabolites is masked due to impurities. Apart from it, the presence of protodioscin, physcion, rutin, liquitrigenin, myricetin in the TBTMF3 of methanol extract could be the reason behind the activity and/or the presence of additional compounds in the extracts. The antioxidant and anti-inflammatory characteristics of the extracts, on the other hand, will need more research in order to understand the mechanisms of action and signaling pathways that are responsible for this activity. In conclusion, *T. terrestris* possesses anti-inflammatory and antioxidant capabilities, as well as the ability to serve as a promising new source of natural chemotherapeutic medications that do not have any side effects. A further investigation must be conducted for the development of effective strategies for *T. terrestris* extraction to obtain bioactive fractions with increased biological activity.

## Figures and Tables

**Table 1:** In vitro antioxidant activities of three different *T. terrestris* extracts compared to control samples.

Assay	DCM	Methanol	70% Aqueous Methanol	Ascorbic Acid	Quercetin
FRAP (Fe mmol/g)	4.10 ± 0.2	35.3 ± 0.5	21.5 ± 1.01	51.0 ± 0.02	62.0 ± 0.02
DPPH (IC_50_ µg/mL)	332.3 ± 2.5	71.4 ± 1.1	141.2 ± 0.01	29.1 ± 0.02	25.4 ± 0.01
H_2_O_2_ (% inhibition)	12.5 ± 0.66	65.3 ± 0.53	34.6 ± 0.83	79 ± 0.02	84 ± 0.05

Values are means ± S.D. DCM; dichloromethane.

**Table 2:** In vitro anti-inflammatory activity of *T. terrestris* crude extracts at 400 µg/mL.

Treatment	Membrane Stabilization	Serum Albumin Denaturation	Egg Albumin Denaturation
	% Inhibition		
Control (phosphate buffer)	NI	NI	NI
Methanol extract (crude extract)	68.5 ***	80.2 ***	75.6 ***
70% aqueous methanol extract (crude extract)	35.9 *	48.3 **	43.2 **
Dichloromethane extract (crude extract)	NA	NA	NA
Diclofenac sodium (standard drug)	89.3 ****	97.8 ****	91.5 ****

Values are means ± S.D. of three measurements. NA, No activity. NI, No inhibition. (* *p* < 0.05, ** *p* < 0.01, *** *p* < 0.001, **** *p* < 0.0001).

**Table 3:** In vitro anticancer activity of the *T. terrestris* crude extracts at 10 µg/mL, 100 µg/mL, 1000 µg/mL (IC_50_ µg/mL).

	MCF-7	HeLa	SK-OV-3	NCI-H522
Methanol	74.1	221.2	89.4	102.1
70% aqueous methanol	176.4	343.1	256.2	441.8
Dichloromethane	NA	NA	NA	NA
Methotrexate (standard drug)	0.87	0.92	0.91	0.88

The values are means ± SE and shown as IC_50_ µg/mL. Each value is expressed as mean of triplicate treatments. HeLa (cervical cancer cell line), MCF-7 (breast cancer cell line), SK-OV-3 (ovary carcinoma), NCI-H522 (lung cancer cell line). NA, No activity.

**Table 4:** Acute and subacute toxicity assessment of *Tribulus terrestris* methanol extract using in vivo model.

Parameters	Control Group	Acute Toxicity (14 days)		Subacute Toxicity (28 days)	
	Normal Saline	2000 mg/kg TBTME	3000 mg/kg TBTME	500 mg/kg TBTME	1000 mg/kg TBTME
Body weight (g)	197.00 ± 8.00	210 ± 3.59	205 ± 4.50	194 ± 10.2	223 ± 11.6
**Organ weights**					
Heart (g)	0.59 ± 0.22	0.66 ± 0.20	0.64 ± 0.05	0.58 ± 0.20	0.73 ± 0.10
Paired Lungs (g)	2.12 ± 1.10	2.22 ± 0.82	2.15 ± 0.80	2.09 ± 1.18	2.42 ± 1.22
Liver (g)	7.89 ± 1.35	8.10 ± 2.90	7.92 ± 1.22	7.79 ± 2.15	8.50 ± 2.90
Spleen (g)	0.44 ± 0.05	0.52 ± 0.01	0.49 ± 0.10	0.42 ± 0.20	0.62 ± 0.30
**Hematological parameters**					
White blood cells (10^5^/µL)	3.35 ± 0.22	4.29 ± 0.30	4.53 ± 0.14	3.69 ± 0.32	3.99 ± 1.89
Neutrophils (%)	38.91 ± 1.10	62.91 ± 2.22	64.01 ± 2.40	60.61 ± 2.34	55.52 ± 27.21
Lymphocytes (%)	43.39 ± 2.30	74.91 ± 4.30	78.60 ± 3.40	63.96 ± 4.93	66.87 ± 33.48
Eosinophils (%)	0.92 ± 0.11	1.46 ± 0.10	1.63 ± 0.08	1.19 ± 0.11	1.70 ± 0.67
Red blood cells (10^6^/µL)	8.90 ± 1.05	16.05 ± 1.20	17.00 ± 1.65	12.91 ± 2.40	17.03 ± 7.80
Hemoglobin (g/dl)	12.92 ± 1.20	26.05 ± 1.95	24.92 ± 2.10	20.02 ± 2.79	22.90 ± 12.49
Hematocrit (%)	46.50 ± 3.60	70.83 ± 3.70	70.43 ± 2.92	59.77 ± 4.24	64.72 ± 30.80
MCV (f/L)	56.20 ± 7.51	99.09 ± 7.50	97.62 ± 5.93	77.66 ± 8.59	85.27 ± 43.41
MCH (pg)	17.91 ± 1.55	28.78 ± 0.25	27.10 ± 2.10	22.90 ± 0.80	25.76 ± 12.18
MCHC (%)	30.93 ± 1.04	43.92 ± 1.10	48.90 ± 0.70	40.06 ± 1.10	43.01 ± 17.80
Platelets (10^5^/µL)	7.45 ± 0.10	11.05 ± 0.40	10.91 ± 1.25	9.12 ± 1.25	11.35 ± 6.20
**Serum biological parameters**					
Total Protein (g/dL)	6.59 ± 2.25	7.31 ± 1.20	8.01 ± 0.90	5.69 ± 2.16	7.50 ± 4.02
Albumin (g/dL)	3.01 ± 1.25	3.70 ± 0.71	3.91 ± 0.45	3.15 ± 0.80	3.72 ± 1.95
Albumin/Globulin ratio	1.80 ± 0.28	3.55 ± 0.20	3.20 ± 0.25	3.60 ± 0.5	3.19 ± 0.75
Lactate Dehydrogenase (U/L)	2230 ± 0.26	3166 ± 271.0	3085.8 ± 214.1	2975.7 ± 310.4	3290.4 ± 231.5
Asparate Transaminase (U/L)	142.0 ± 271.0	191.3 ± 10.10	187.4 ± 7.98	175.1 ± 11.57	179.2 ± 29.34
Alanine Transaminase (U/L)	25.30 ± 10.10	59.51 ± 5.50	57.66 ± 4.35	48.47 ± 6.30	53.80 ± 7.96
Alkaline Phosphatase (U/L)	379.0 ± 9.10	418.0 ± 13.10	417.0 ± 10.82	391.2 ± 16.91	397.5 ± 19.02
Total bilirubin (mg/dL)	0.34 ± 0.12	0.70 ± 0.10	1.42 ± 0.04	0.39 ± 0.06	2.19 ± 1.29
Creatinine (mg/dL)	1.91 ± 0.09	3.14 ± 0.20	3.43 ± 0.24	2.59 ± 0.07	3.26 ± 0.79
Uric Acid (mg/dl)	0.91 ± 12.3	1.62 ± 0.12	2.19 ± 0.04	1.36 ± 0.12	2.91 ± 35.02
Total Cholesterol (mg/dl)	50.91 ± 3.15	85.00 ± 4.20	84.01 ± 2.90	70.12 ± 6.13	77.02 ± 67.31
Triglycerides (mg/dl)	117.2 ± 5.12	189.2 ± 8.12	182.6 ± 9.03	155.0 ± 9.71	154.2 ± 80.19
Potassium (mmol/L)	2.54 ± 1.42	5.39 ± 2.20	5.53 ± 2.43	4.54 ± 0.13	5.19 ± 1.11
Chloride (mmol/L)	70.24 ± 18.31	144.2 ± 19.22	145.2 ± 14.70	111.4 ± 21.81	152.0 ± 12.40
Sodium (mmol/L)	136.1 ± 17.20	190.4 ± 17.22	192.0 ± 13.42	150.0 ± 21.52	183.0 ± 64.61

TBTME, *Tribulus terrestris* methanol extract. MCV, Mean corpuscular volume. MCH, Mean corpuscular hemoglobin. MCHC, Mean corpuscular hemoglobin concentration.

**Table 5:** Biological activities of *T. terrestris* liquid–liquid partitioned fractions compared to control samples.

Assays	Fraction (A)	Fraction (B)	Fraction (C)	Ascorbic Acid	Quercetin	Diclofenac Sodium	Methotrexate
**Antioxidant activity**							
FRAP (mmol/g)	NA	45.2 ± 0.1	17.2 ± 0.10	50.9 ± 0.20	61.9 ± 0.05	-	-
DPPH (IC_50_ µg/mL)	NA	56.2 ± 1.1	91.9 ± 0.01	30.2 ± 0.05	24.9 ± 0.10	-	-
H_2_O_2_ (%)	NA	68.0 ± 0.2	32.1 ± 0.5	79.0 ± 0.02	84.0 ± 0.05	-	-
**Anti-inflammatory activity (% inhibition at 400 µg/mL)**							
Heat-induced hemolysis	NA	74.1 ***	36.9 ± 0.10 *	-	-	89.3 ****	-
Egg albumin denaturation	NA	77.9 ***	39.6 ± 0.2 *	-	-	91.5 ****	-
Serum albumin denaturation	NA	83.5 ***	52.1 ± 0.10 *	-	-	97.8 ****	-
**Anti-cancer activity (IC_50_ µg/mL)**							
MCF-7 Breast cancer	NA	65.2	122.8	-	-	-	0.80
HeLa Cervical cancer	NA	223.6	NA	-	-	-	0.92
SK-OV-3 Ovary carcinoma	NA	81.3	231.8	-	-	-	0.92
NCI-H522 Lung cancer	NA	111.9	174.9	-	-	-	0.88

Values are means ± S.D., FRAP, Ferric reducing antioxidant power assay. DPPH, 2,2-diphenyl-1-picrylhydrazyl. H_2_O_2_, Hydrogen peroxide. NA, No activity. (* *p* < 0.05, *** *p* < 0.001, **** *p* < 0.0001)

**Table 6:** Biological activities of RP–HPLC separated sub-fractions of the *T. terrestris* methanol extract.

Assays	TBTMF1	TBTMF2	TBTMF3	TBTMF4	Ascorbic Acid	Quercetin	Diclofenac Sodium	Methotrexate
**Antioxidant activity**								
FRAP (mmol/g)	NA	26.1 ± 0.3	49.1 ± 0.1	38.8 ± 1.1	51 ± 0.02	62 ± 0.02	-	-
DPPH (IC_50_ µg/mL)	NA	96.6 ± 0.2	41.9 ± 1.1	104.6 ± 0.1	29.1 ± 0.02	25.4 ± 0.01	-	-
H_2_O_2_ (%)	NA	28.1 ± 1.1	71.2 ± 0.5	17.3 ± 0.2	79 ± 0.02	84 ± 0.05	-	-
**Anti-inflammatory activity (% inhibition at 400 µg/mL)**								
Membrane stabilization	NA	33.2	76.1	29.5	-	-	89.3	-
Egg albumin denaturation	NA	37.8	81.9	30.8	-	-	91.5	-
Serum albumin denaturation	NA	44.8	85.2	34.5	-	-	97.8	-
**Anticancer activity (IC_50_ µg/mL)**								
Breast cancer (MCF-7)	NA	281.4	43.2	331.9	-	-	-	0.87
Cervical cancer (HeLa)	NA	441.9	142.5	NA	-	-	-	0.92
Ovary carcinoma (SK-OV-3)	NA	NA	88.4	241.9	-	-	-	0.96
Lung cancer (NCI-H522)	NA	321.4	298.6	NA	-	-	-	0.88

TBTMF. *T. terrestris* methanol fraction, - Not evaluated.

**Table 7:** Quantification of rutin, protodioscin, and myricetin from *T. terrestris* *RP–HPLC* sub-fraction TBTMF3.

Compound Name	LOD (µg/mg)	LOQ (µg/mg)	*r* ^2^	R_t_ min	Concentration (µg/mg)
Rutin	1.70	4.90	0.9998	4.9	2.19
Protodioscin	1.10	3.20	0.9986	5.1	11.2
Myricetin	1.90	5.60	0.9999	22.3	4.2

Shown are mean values of three measurements, Limit of quantification (LOQ), Limit of detection (LOD), Coefficient of regression (*r*^2^), time of retention (R_t_), *Tribulus Terrestris* methanolic fraction 3 (TBTMF3).

## Data Availability

All data related to research presented in the manuscript.

## References

[1] Rodriguez V.L., Davoudian T. (2016). Clinical Measurement of Pain, Opioid Addiction, and Functional Status. Treating Comorbid Opioid Use Disorder in Chronic Pain.

[2] Shirzad H., Rafieian-Kopaei M. (2018). Recent findings in molecular basis of inflammation and anti-inflammatory plants. Curr. Pharm. Des..

[3] Sung H., Ferlay J., Siegel R.L., Laversanne M., Soerjomataram I., Jemal A., Bray F. (2021). Global Cancer Statistics 2020: GLOBOCAN Estimates of Incidence and Mortality Worldwide for 36 Cancers in 185 Countries. CA Cancer J. Clin..

[4] Charepalli V., Reddivari L., Vadde R., Walia S., Radhakrishnan S., Vanamala J.K.P. (2016). *Eugenia jambolana* (Java Plum) fruit extract exhibits anti-cancer activity against early stage human hct-116 colon cancer cells and colon cancer stem cells. Cancers.

[5] Stanković N., Mihajilov-Krstev T., Zlatković B., Stankov-Jovanović V., Mitić V., Jović J., Čomić L., Kocić B., Bernstein N. (2016). Antibacterial and antioxidant activity of traditional medicinal plants from the Balkan Peninsula. NJAS—Wagening. J. Life Sci..

[6] Cragg G.M., Newman D.J. (2005). Plants as a source of anti-cancer agents. J. Ethnopharmacol..

[7] Samuelsson G. (2004). Drugs of Natural Origin. A Textbook of Pharmacognosy.

[8] Debjit B., Pawan D., Margret C., Kumar K.P.S. (2009). Herbal drug toxicity and safety evaluation of traditional medicines. Arch. Appl. Sci. Res..

[9] Thelingwani R., Masimirembwa C. (2014). Evaluation of herbal medicines: Value addition to traditional medicines through metabolism, pharmacokinetic and safety studies. Curr. Drug Metabol..

[10] Chhatre S., Nesari T., Kanchan D., Somani G., Sathaye S. (2014). Phytopharmacological overview of *Tribulus terrestris*. Pharmacogn. Rev..

[11] Neychev V., Mitev V. (2015). Pro-sexual and androgen enhancing effects of *Tribulus terrestris* L.: Fact or fiction. J. Ethnopharmacol..

[12] Chinese Pharmacopoeia Commission (2015). Chinese Pharmacopoeia.

[13] Mohammed M.S., Alajmi M.F., Alam P., Ali M.M., Mahmoud A.M., Ahmed W.J. (2014). Chromatographic finger print analysis of anti–inflammatory active extract fractions of aerial parts of *Tribulus terrestris* by HPTLC technique. Asian Pac. J. Trop. Biomed..

[14] Akram M., Asif H.M., Akhtar N., Shah P.A., Uzair M., Shaheen G., Shah S.A. (2011). *Tribulus terrestris* Linn.: A review article. J. Med. Plant Res..

[15] Nam J., Jung H.W., Chin Y.-W., Kim W.K., Bae H.S. (2016). Modulatory effects of the fruits of *Tribulus terrestris* L. on the function of atopic dermatitis-related calcium channels, Orai1 and TRPV3. Asian Pac. J. Trop. Biomed..

[16] Zhang N., Jia Y., Chen G., Cabrales P., Palmer A.F. (2011). Biophysical properties and oxygenation potential of high-molecular-weight glutaraldehyde-polymerized human hemoglobins maintained in the tense and relaxed quaternary states. Tissue Eng. Part A.

[17] Kim H.J., Kim J.C., Min J.S., Kim M.J., Kim J.A., Kor M.H., Ahn J.K. (2011). Aqueous extract of *Tribulus terrestris* Linn induces cell growth arrest and apoptosis by down-regulating NF-κB signaling in liver cancer cells. J. Ethnopharmacol..

[18] Gopinath V., MubarakAli D., Priyadarshini S., Priyadharsshini N.M., Thajuddin N., Velusamy P. (2012). Biosynthesis of silver nanoparticles from *Tribulus terrestris* and its antimicrobial activity: A novel biological approach. Colloids Surf. B Biointerfaces.

[19] Sailaja K., Shivaranjani V.L., Poornima H., Rahamathulla S., Devi K.L. (2013). Protective effect of *Tribulus terrestris* L. fruit aqueous extracton lipid profile and oxidative stress in isoproterenol induced myocardial necrosis in male albino Wistar rats. EXCLI J..

[20] Fatima L., Sultana A. (2017). Efficacy of *Tribulus terrestris* L. (fruits) in menopausal transition symptoms: A randomized placebo controlled study. Adv. Integr. Med..

[21] Ma Y., Guo Z., Wang X. (2015). *Tribulus terrestris* extracts alleviate muscle damage and promote anaerobic performance of trained male boxers and its mechanisms: Roles of androgen, IGF-1, and IGF binding protein-3. J. Sport Health Sci..

[22] Kang S.Y., Jung H.W., Nam J.H., Kim W.K., Kang J.S., Kim Y.H., Bae H.S. (2017). Effects of the fruit extract of *Tribulus terrestris* on skin inflammation in mice with oxazolone-induced atopic dermatitis through regulation of calcium channels, orai-1 and TRPV3, and mast cell activation. Evid. Based Complement. Altern..

[23] Singleton V.L., Rossi J.A. (1965). Colorimetry of total phenolics with phosphomolybdic-phosphotungstic acid reagents. Am. J. Enol. Vitic..

[24] Pękal A., Pyrzynska K. (2014). Evaluation of aluminium complexation reaction for flavonoid content assay. Food Anal. Methods.

[25] Alara O., Abdurahman N., Mudalip S.A., Olalere O. (2019). Effect of drying methods on the free radicals scavenging activity of *Vernonia amygdalina* growing in Malaysia. J. King Saud Univ. Sci..

[26] Zahin M., Aqil F., Ahmad I. (2010). Broad spectrum antimutagenic activity of antioxidant active fraction of *Punica granatum* L. peel extracts. Mutat. Res. Toxicol. Environ. Mutagen..

[27] Ruch R.J., Cheng S.J., Klaunig J.E. (1989). Prevention of cytotoxicity and inhibition of intercellular communication by antioxidant catechins isolated from Chinese green tea. Carcinogenesis.

[28] Sadique J., Al-Rqobahs W.A., Bughaith E.I., Gindi A.R. (1989). The bioactivity of certain medicinal plants on the stabilization of RBC membrane system. Fitoterapia.

[29] Sakat S., Juvekar A.R., Gambhire M.N. (2010). In vitro antioxidant and anti-inflammatory activity of methanol extract of *Oxalis corniculata* Linn. Int. J. Pharm. Pharm. Sci..

[30] Mizushima Y., Kobayashi M. (1968). Interaction of anti-inflammatory drugs with serum proteins, especially with some biologically active proteins. J. Pharm. Pharmacol..

[31] Morris C.J. (2003). Carrageenan-induced paw edema in the rat and mouse. Methods Mol. Biol..

[32] Brownlee G. (1950). Effect of deoxycortone and ascorbic acid on formaldehyde-induced arthritis in normal and adrenalectomised rats. Lancet.

[33] OECD (2008). OECD Guideline for Testing of Chemicals. Repeated Dose 28-day Oral Toxicity in Rodents, Test No. 407.

[34] OECD (2001). OECD Guidelines for Testing of Chemicals: Acute Oral Toxicity—Acute Toxic Class Method. Test No. 423, Adopted 22nd March 1996, and Revised Method Adopted 17th December 2001.

[35] Steinmann D., Ganzera M. (2011). Recent advances on HPLC/MS in medicinal plant analysis. J. Pharm. Biomed. Anal..

[36] Gates P.J., Lopes N.P. (2012). Characterisation of Flavonoid Aglycones by Negative Ion Chip-Based Nanospray Tandem Mass Spectrometry. Int. J. Anal. Chem..

[37] Rahman H., Khan I., Hussain A., Shahat A.A., Tawab A., Qasim M., Adnan M., Al-Said M.S., Ullah R., Khan S.N. (2018). *Glycyrrhiza glabra* HPLC fractions: Identification of aldehydo isoophiopogonone and liquirtigenin having activity against multidrug resistant bacteria. BMC Complement. Altern. Med..

[38] Chen G., Li X., Saleri F., Guo M. (2016). Analysis of flavonoids in *Rhamnus davurica* and its antiproliferative activities. Molecules.

[39] Angelova S., Gospodinova Z., Krasteva M., Antov G., Lozanov V., Markov T., Bozhanov S., Georgieva E., Mitev V. (2013). Antitumor activity of Bulgarian herb *Tribulus terrestris* L. on human breast cancer cells. J. Biosci. Biotechnol..

[40] Said I.H., Shah R.L., Ullrich M.S., Kuhnert N. (2016). Quantification of microbial uptake of quercetin and its derivatives using an UHPLC-ESI-QTOF mass spectrometry assay. Food Funct..

[41] Tian C., Chang Y., Zhang Z., Wang H., Xiao S., Cui C., Liu M. (2019). Extraction technology, component analysis, antioxidant, antibacterial, analgesic and anti-inflammatory activities of flavonoids fraction from *Tribulus terrestris* L. leaves. Heliyon.

[42] Naz R., Ayub H., Nawaz S., Islam Z.U., Yasmin T., Bano A., Wakeel A., Zia S., Roberts T.H. (2017). Antimicrobial activity, toxicity and anti-inflammatory potential of methanolic extracts of four ethnomedicinal plant species from Punjab, Pakistan. BMC Complement. Altern. Med..

[43] Amorati R., Valgimigli L. (2018). Methods to measure the antioxidant activity of phytochemicals and plant extracts. J. Agric. Food Chem..

[44] Arshad M., Rahman A., Qayyum A., Hussain K., Khan M.A., Hussain T., Abbas M., Shar G.A., Zahoor M.K., Nazir A. (2020). Environmental applications and bio-profiling of *tribulus terrestris*: An ecofriendly approach. Pol. J. Environ. Stud..

[45] Kumari C.S., Yasmin N., Hussain M.R., Babuselvam M. (2015). In vitro anti-inflammatory and anti-arthritic property of *Rhizopora mucronata* leaves. Intern. J. Pharm. Sci. Res..

[46] Ghareeb D.A., El Ahwany A., El-Mallawany S., Saif A.A. (2014). In vitro screening for anti-acetylcholiesterase, anti-oxidant, anti-glucosidase, anti-inflammatory and anti-bacterial effect of three traditional medicinal plants. Biotechnol. Biotechnol. Equip..

[47] Opie E.L. (1962). On the relation of necrosis and inflammation to denaturation of proteins. J. Exp. Med..

[48] Williams L.A.D., O’Connar A., Latore L., Dennis O., Ringer S., Whittaker J.A., Conrad J., Vogler B., Rosner H., Kraus W. (2008). The in vitro anti-denaturation effects induced by natural products and non-steroidal compounds in heat treated (immunogenic) bovine serum albumin is proposed as a screening assay for the detection of anti-inflammatory compounds, without the use of animals, in the early stages of the drug discovery process. West Indian Med. J..

[49] Huang Y.-C., Hwang T.-L., Chang C.-S., Yang Y.-L., Shen C.-N., Liao W.-Y., Chen S.-C., Liaw C.-C. (2009). Anti-inflammatory Flavonoids from the Rhizomes of *Helminthostachys zeylanica*. J. Nat. Prod..

[50] Anosike C.A., Igboegwu O.N., Nwodo O.F.C. (2018). Antioxidant properties and membrane stabilization effects of methanol extract of *Mucuna pruriens* leaves on normal and sickle erythrocytes. J. Tradit. Complement. Med..

[51] Sudheendran A., Shajahan M.A. (2017). Anti-inflammatory activity of root and fruit of gokshura (*Tribulus terrestris* Linn.) In albino rats. Int. J. Ayurveda Phar. Res..

[52] Ahmad S., Ansari J.A., Jamil M., Qamruzzama Q. (2012). Wound healing potential of methanolic extract of *Tribulus terrestris* L. Fruits. J. Drug Deliv. Ther..

[53] Baburao B., Rajyalakshmi G., Venkatesham A., Kiran G., Shyamsunder A., Gangarao B. (2009). Anti-inflammatory and antimicrobial activities of methanolic extract of *Tribulus terrestris* Linn plant. Int. J. Chem. Sci..

[54] Ahmad N., Qamar M., Yuan Y., Nazir Y., Wilairatana P., Mubarak M.S. (2022). Dietary Polyphenols: Extraction, Identification, Bioavailability, and Role for Prevention and Treatment of Colorectal and Prostate Cancers. Molecules.

[55] Bedir E., Khan I.A., Walker L.A. (2002). Biologically active steroidal glycosides from *Tribulus terrestris*. Pharmazie.

[56] Tan Y.-J., Ren Y.-S., Gao L., Li L.-F., Cui L.-J., Li B., Li X., Yang J., Wang M.-Z., Lv Y.-Y. (2018). 28-Day Oral Chronic Toxicity Study of Arctigenin in Rats. Front. Pharmacol..

[57] Das N., Goshwami D., Hasan S., Raihan S.Z. (2015). Evaluation of acute and subacute toxicity induced by methanol extract of *Terminalia citrina* leaves in Sprague Dawley rats. J. Acute Dis..

[58] Nalawade S.A., Pingale S.S., Chaskar M.G. (2019). Study of acute toxicity of *Tribulus terristris*. JCPR.

[59] Mukhi S., Bose A., Das D.K., Panda S.K., Mohapatra D., Latha S., Balaraman A.K. (2021). Acute and Sub-acute Toxicity study of Amrtadi Churna. Res. J. Pharm. Technol..

[60] Raoofi A., Khazaei M., Ghanbari A. (2015). Protective effect of hydroalcoholic extract of *Tribulus terrestris* on cisplatin induced renal tissue damage in mal e mice. Int. J. Prev. Med..

[61] Malheiros A., Filho V.C., Schmitt C.B., Yunes R.A., Escalante A., Svetaz L., Zacchino S., Monache F.D. (2005). Antifungal activity of drimane sesquiterpenes from *Drimys brasiliensis* using bioassay-guided fractionation. J. Pharm. Pharm. Sci..

[62] Zhang X., Han F., Gao P., Yu D., Liu S. (2007). Bioassay-guided fractionation of antifertility components of castorbean (*Ricinus communis* L.) seed extracts. Nat. Prod. Res..

[63] Ediriweera M.K., Tennekoon K.H., Samarakoon S.R., Thabrew I., De Silva E.D. (2016). A study of the potential anticancer activity of *Mangifera zeylanica* bark: Evaluation of cytotoxic and apoptotic effects of the hexane extract and bioassay-guided fractionation to identify phytochemical constituents. Oncol. Lett..

[64] Qamar M., Akhtar S., Ismail T., Yuan Y., Ahmad N., Tawab A., Ismail A., Barnard R.T., Cooper M.A., Blaskovich M.A. (2021). *Syzygium cumini*(L.), Skeels fruit extracts: In vitro and in vivo anti-inflammatory properties. J. Ethnopharmacol..

[65] Qamar M., Akhtar S., Barnard R.T., Sestili P., Ziora Z.M., Lazarte C.E., Ismail T. (2022). Antiinflammatory and Anticancer Properties of *Grewia asiatica* Crude Extracts and Fractions: A Bioassay-Guided Approach. BioMed Res. Int..

[66] Abbas M.W., Hussain M., Qamar M., Ali S., Shafiq Z., Wilairatana P., Mubarak M.S. (2021). Antioxidant and Anti-Inflammatory Effects of *Peganum harmala* Extracts: An In Vitro and In Vivo Study. Molecules.

[67] Liu J.Y., Hou Y.L., Cao R., Qiu H.X., Cheng G.H., Tu R., Wang L., Zhang J.L., Liu D. (2017). Protodioscin ameliorates oxidative stress, inflammation and histology outcome in Complete Freund’s adjuvant induced arthritis rats. Apoptosis.

[68] Nafees S., Rashid S., Ali N., Hasan S.K., Sultana S. (2015). Rutin ameliorates cyclophosphamide induced oxidative stress and inflammation in Wistar rats: Role of NFκB/MAPK pathway. Chem. Interact..

[69] Wang S.J., Tong Y., Lu S., Yang R., Liao X., Xu Y.F., Li X. (2010). Anti-inflammatory activity of myricetin isolated from *Myrica rubra* Sieb. et Zucc. leaves. Planta Med..

[70] Kim Y.W., Zhao R.J., Park S.J., Lee J.R., Cho I.J., Yang C.H., Kim S.G. (2008). Anti-inflammatory effects of liquiritigenin as a consequence of the inhibition of NF-κB-dependent iNOS and proinflammatory cytokines production. J. Cereb. Blood Flow Metab..

[71] Jiao D., Zhang X.D. (2016). Myricetin suppresses p21-activated kinase 1 in human breast cancer MCF-7 cells through downstream signaling of the β-catenin pathway. Oncol Rep..

[72] Sajedi N., Homayoun M., Mohammadi F., Soleimani M. (2020). Myricetin exerts its apoptotic effects on MCF-7 breast cancer cells through evoking the BRCA1-GADD45 pathway. Asian Pac. J. Cancer Prev..

[73] Liang F., Zhang H., Gao H., Cheng D., Zhang N., Du J., Yue J., Du P., Zhao B., Yin L. (2020). Liquiritigenin decreases tumorigenesis by inhibiting DNMT activity and increasing BRCA1 transcriptional activity in triple-negative breast cancer. Exp. Biol. Med..

[74] Iriti M., Kubina R., Cochis A., Sorrentino R., Varoni E.M., Kabała-Dzik A., Azzimonti B., Dziedzic A., Rimondini L., Wojtyczka R.D. (2017). Rutin, a quercetin glycoside, restores chemosensitivity in human breast cancer cells. Phytother. Res..

[75] Elsayed H.E., Ebrahim H.Y., Mohyeldin M.M., Siddique A.B., Kamal A.M., Haggag E., El Sayed K.A. (2017). Rutin as a novel c-Met inhibitory lead for the control of triple negative breast malignancies. Nutr. Cancer.

[76] Dinchev D., Janda B., Evstatieva L., Oleszek W., Aslani M.R., Kostova I. (2008). Distribution of steroidal saponins in *Tribulus terrestris* from different geographical regions. Phytochemistry.

[77] Ivanova S., Ivanov K., Mladenov R., Papanov S., Ivanova S., Obreshkova D., Atanasov P.P.V., Petkova V. (2016). Food supplements with anabolic and androgenic activity-UHPLC analysis of food additives, containing *Tribulus terrestris* extract. World J. Pharma. Res..

[78] Kumar A. (2012). Comparative and quantitative determination of quercetin and other flavonoids in North Indian populations of *Tribulus terrestris* Linn, by HPLC. Int. J. Pharm. Bio. Sci..

