# Correction: Abbas et al. Bioactive Compounds, Antioxidant, Anti-Inflammatory, Anti-Cancer, and Toxicity Assessment of *Tribulus terrestris*—In Vitro and In Vivo Studies. *Antioxidants* 2022, *11*, 1160

**DOI:** 10.3390/antiox13040471

**Published:** 2024-04-17

**Authors:** Malik Waseem Abbas, Mazhar Hussain, Saeed Akhtar, Tariq Ismail, Muhammad Qamar, Zahid Shafiq, Tuba Esatbeyoglu

**Affiliations:** 1 Institute of Chemical Sciences, Bahauddin Zakariya University, Multan 60800, Pakistan; wasimchemist229@gmail.com (M.W.A.); zahidshafiq@bzu.edu.pk (Z.S.); 2 Institute of Food Science and Nutrition, Bahauddin Zakariya University, Multan 60800, Pakistan; saeedakhtar@bzu.edu.pk (S.A.); tariqismail@bzu.edu.pk (T.I.); 3 Institute of Food Science and Human Nutrition, Gottfried Wilhelm Leibniz University Hannover, Am Kleinen Felde 30, 30167 Hannover, Germany

In the original publication [1], there was a mistake in Figure 2 as published. We usually zoom in to the pictures to ensure they are different; however, the same picture was mistakenly uploaded in this frame. Normal kidney tissue and treated kidney tissue appeared to be the same. The corrected Figure 2 appears below.

The authors state that the scientific conclusions remain unaffected. This correction was approved by the Academic Editor. The original publication has also been updated.

## Figures and Tables

**Figure 2:**
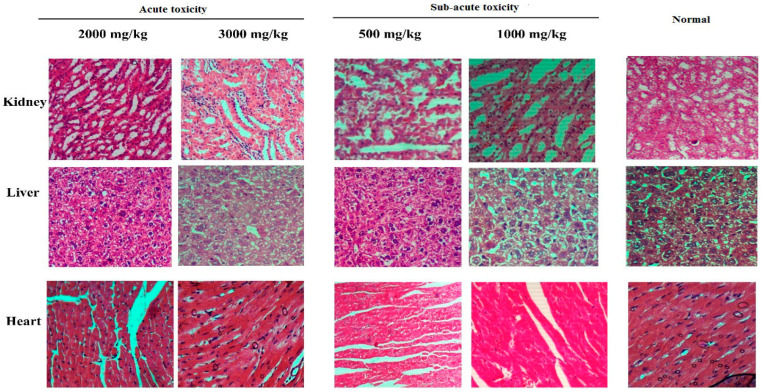
Histopathology results of acute and subacute toxicity of *T. terrestris* methanol extract.
